# Prognostic impact of programed cell death-1 (PD-1) and PD-ligand 1 (PD-L1) expression in cancer cells and tumor infiltrating lymphocytes in colorectal cancer

**DOI:** 10.1186/s12943-016-0539-x

**Published:** 2016-08-24

**Authors:** Yaqi Li, Lei Liang, Weixing Dai, Guoxiang Cai, Ye Xu, Xinxiang Li, Qingguo Li, Sanjun Cai

**Affiliations:** 1 Department of Colorectal Surgery, Fudan University Shanghai Cancer Center, 270 Dong’an Road, Shanghai, 20032 China; 2 Department of Oncology, Shanghai Medical College, Fudan University, 270 Dong’an Road, Shanghai, 20032 China

**Keywords:** PD-1, PD-L1, Tumor infiltrating lymphocytes, Colorectal cancer, The cancer genome atlas, Prognosis

## Abstract

**Background:**

Colorectal cancer (CRC) is 3rd most commonly diagnosed cancer in males and the second in females. PD-1/PD-L1 axis, as an immune checkpoint, is up-regulated in many tumors and their microenvironment. However, the prognostic value of PD-1/PD-L1 in CRC remains unclear.

**Methods:**

The Cancer Genome Atlas (TCGA) database (*N* = 356) and Fudan University Shanghai Cancer Center (FUSCC) cohort of patients (*N* = 276) were adopted to analyze the prognostic value of PD-L1 in colorectal tumor cells (TCs) and of PD-1 in tumor infiltrating cells (TILs) for CRC. Subgroup analyses were conducted in FUSCC cohort according to patients’ status of mismatch repair.

**Results:**

In TCGA cohort, the cut-off values of PD-1 and PD-L1 expression were determined by X-tile program, which were 4.40 and 2.92, respectively. Kaplan-Meier analysis indicated that higher PD-1 and PD-L1 expressions correlated with better OS (*P* = 0.032 and *P* = 0.002, respectively). In FUSCC cohort, expressions of PD-1 on TILs and PD-L1 on TCs were analyzed separately by immunohistochemistry (IHC) staining based on a TMA sample (*N* = 276) and revealed that both TILs-PD-1 and TCs-PD-L1 were associated with OS (*P* = 0.006 and *P* = 0.002, respectively) and DFS (*P* = 0.025 and *P* = 0.004, respectively) of CRC patients. Multivariate Cox regression analysis indicated TILs-PD-1 was an independent prognostic factor both for OS and DFS of CRC patients (*P* < 0.05). Subgroup analyses showed that TILs-PD-1 was an independent prognostic factor for both OS and DFS in CRC patients in MSS-proficient subgroup (*P* < 0.05), while neither of them correlated with OS or DFS in MSS-deficient subgroup (*P* > 0.05).

**Conclusions:**

Higher expressions of PD-1 and PD-L1 correlates with better prognosis of CRC patients. TILs-PD-1 is an independent prognostic factor for OS and DFS of CRC patients, especially for MMR-proficient subgroup.

**Electronic supplementary material:**

The online version of this article (doi:10.1186/s12943-016-0539-x) contains supplementary material, which is available to authorized users.

## Background

Colorectal cancer (CRC) is the third most commonly diagnosed cancer in males and the second in females, accounting for approximately 9.7 % of total cancer cases and approximately 8.5 % of cancer deaths [1]. A considerable proportion of CRC patients develop local recurrence and distant metastasis within 5 years after surgical treatment. Immunotherapy has reached center stage in the field of second-line therapy in oncology treatment, and anti-PD-1 therapy has shown objective responses in variety of human malignancies, including melanoma, non-small cell lung cancer and renal cell carcinoma [2]. However, only microsatellite instable (MSI) subset of CRC seems to be a good candidate for checkpoint blockage immunotherapy [3] and the mechanisms are still controversial.

Tumor-infiltrating lymphocytes (TILs) are widely considered as reflection of primary host immune response against solid tumors. Evidence has shown that tumor infiltration by activated CD8^+^ cytotoxic T lymphocytes correlates with better survival of CRC patients [4]. PD-1/PD-L1 axis, as an immune checkpoint, is up-regulated in many tumors and their microenvironment, and is a negative feedback system that represses Th1 cytotoxic immune responses [5]. The engagement of PD-1 by its ligands (PD-L1 and PD-L2) induces down-regulation of antigen-stimulated lymphocyte proliferation and cytokine (such as IFN-γ and IL-2) production, resulting in lymphocyte deletion and in the induction of immunological tolerance [3, 6–8]. Compared to PD-L2, which can be detected only in activated dendritic cells (DC) and macrophages, PD-L1 is constitutively expressed by T and B cells, DC and macrophages [7, 8], and it is also expressed in additional cell types, such as endothelial, pancreatic and muscle cells [9].

The expression of PD-L1 in tumor cells (TCs) has been validated as a predictive marker for tumor response to anti-PD-1 or PD-L1 immunotherapy in different malignancies [2, 10–12]. However, the prognostic value of PD-1 and PD-L1 expression in different cancers are still controversial. In the present study, we used 356 cases in The Cancer Genome Atlas (TCGA) database and a tissue microarray (TMA) including 276 well-documented, clinically annotated CRC specimens in Fudan University Shanghai Cancer Center (FUSCC) to investigate the expression of PD-1 and PD-L1 in CRC and their clinical significance.

## Methods

### The cancer genome atlas (TCGA) database

PD-1 and PD-L1 expression in patients of CRC and clinical data of TCGA database are available from the website of Cancer Genomics Browser of University of California Santa Cruz (https://genome-cancer.ucsc.edu/). In total, 356 primary CRC tumors from patients with detailed PD-1 and PD-L1 expression data were chosen from the updated TCGA database according to parameters defined in a previous study [13]. Only patients with fully characterized tumors, intact overall survival (OS), complete RNAseq information, and patients without pretreatment were included. Clinicopathological characteristics, including age, gender, tumor location, historical type, TNM stage, venous invasion, extent of TILs, microsatellite (MS) status, pretreatment CEA and overall survival were collected.

### Tissue microarray (TMA) construction and clinicopathological features

The TMA used for this study includes 276 unselected, non-consecutive, primary, and sporadic CRCs treated between January 2007 and November 2009 in Fudan University Shanghai Cancer Center (FUSCC). Construction of this TMA has been previously described in detail [14]. Briefly, formalin-fixed, paraffin-embedded tissue blocks from resected CRC were obtained. Tissue cylinders with a 0.6 mm diameter were punched from representative tissue areas of each donor tissue block and brought into one recipient paraffin block (30 × 25 mm). Each TMA spot included at least 50 % tumor cells. The histological types were confirmed by experienced pathologists.

Patients’ demographic and clinicopathological variables, including age, sex, primary site, histological type, TNM stage, pathological grade, venous/nervous invasion, regional lymph node retrieval, MMS status, pretreatment CEA level, treatment type et al., were retrieved from the FUSCC database. All patients were restaged according to the 7th edition of the TNM-UICC/AJCC classification. Venous/nervous invasion was identified by experienced pathologists using hemotoxylin/eosin (HE) staining. The regimens used varied because of long duration of data collection. So, we simply classify patients into two groups according to whether patients had received adjuvant chemotherapy or not. Patients were followed up regularly according to NCCN guidelines. Physical examination and serum tumor biomarkers, including CEA, were performed every 3 to 6 months for the first 2 years, 6 months within the third to fifth year, then annually. Chest/abdominal/pelvis CTs were performed annually for up to 5 years, and colonoscopy was performed 1st year after treatment and repeated in 3rd year if no advanced adenoma was found and then every 5 years. As this study described the prognosis of patients with CRC, analysis of OS and DFS were ascertained. The OS was defined as the time from treatment to death from any cause, and the DFS was defined as the time from treatment to the first recurrence or death. The survival data was provided by Clinical Statistics Center of FUSCC, relying on the hospital medical records follow-up platform or contacts with patients by phone or email. Patients who were alive at last follow-up were censored for analysis.

### Immunohistochemistry (IHC)

Immunohistochemical (IHC) staining was performed according to standard protocol. Briefly, paraffin-embedded samples were cut into 4 μm sections and placed on polylysine-coated slides. Paraffin sections were baked overnight at 58 °C, de-paraffinized in xylene, rehydrated through graded ethanol, quenched for endogenous peroxidase activity in 0.3 % hydrogen peroxide at 37 °C for 15 min, and processed for antigen retrieval by high pressure cooking in citrate antigen retrieval solution (pH = 6.0) for about 10 min for PD-L1 and EDTA antigen retrieval solution (pH = 8.0) for about 4 min for PD-1. Sections were incubated at 37 °C for 1.5 h with rabbit monoclonal antibodies against PD-1 (1:100, ab137132, Abcam, Cambridge, MA, USA) and PD-L1 (1:50, ab174838, Abcam, Cambridge, MA, USA) in a moist chamber. Immunostaining was performed using the EnVision^+^System-HRP (AEC) (K4005, Dako, Glostrup, Denmark), which resulted in a brown-colored precipitate at the antigen site. Subsequently, sections were counterstained with hematoxylin (Sigma-Aldrich, St Louis, MO, USA) and mounted in a non-aqueous mounting medium. All runs included a no primary antibody control.

### Evaluation of PD-1 and PD-L1 expression TCs and TILs in FUSCC cohort

The immunohistochemically stained tissue sections were scored separately by two pathologists blinded to the clinicopathological parameters. The staining intensity was scored as 0 (negative), 1 (weak), 2 (medium) or 3 (strong). Extent of staining was scored as 0 (<5 %), 1(5–25 %), 2 (26–50 %), 3 (51–75 %) and 4 (>75 %) according to the percentages of the positive staining areas in relation to the whole carcinoma area. Scores for staining intensity and percentage positivity of cells were then multiplied to generate the immunoreactivity score (IRS) for each case. Samples having a final staining score of ≤ 4 were considered to be low and those with score of > 4 were considered to be high.

The extent of TILs was assessed in HE stained TMA preparations using a 4-degree scale on the visual estimation and recorded as 0 (absent), 1 (<30 %), 2 (30 %–60 %) and 3 (>60 %). Samples with a score of 0 or 1 were considered negative and those with a score of 2 or 3 were considered positive. The expression of PD-1 in TILs by IHC method was evaluated using IRS mentioned above.

### Microsatellite (MS) status and mismatch repair (MMR) status

In the TCGA cohort, MSI was evaluated by exome sequence analysis [15]. Based on the assay, 243 CRCs in the TCGA cohort could be classified as MSS and 113 as MSI. In the FUSCC cohort, CRCs were stratified according to DNA mismatch repair (MMR) status as described elsewhere [16, 17]. Briefly, MMR-proficient tumors were defined as those simultaneously expressing MutL homolog 1 (MLH1), MutS homolog 2 (MSH2) and MutS homolog 6 (MSH6), while MMR-deficient tumors were defined as those lacking expression of at least one of these markers. Based on these features, 176 CRCs in FUSCC cohort could be classified as MMR-proficient and 100 as MMR-deficient.

### Statistical analysis

Statistical evaluation was conducted with SPSS 22.0 (SPSS Inc., Chicago, IL, USA) and GraphPad Prism v.6 (La Jolla, CA, USA). X-tile 3.6.1 software [18] (Yale University, New Haven, CT, USA) was used to determine the optimal cut-off values for PD-1 and PD-L1 expression in TCGA cohort. Chi-square test was used to analyze the relationship between clinicopathological parameters and PD-l, PD-L1 expressions. Survival analysis was performed using the Kaplan-Meier method and Cox regression model. *P* < 0.05 was considered statistically significant. All confidence intervals (CIs) were stated at the 95 % confidence level.

## Results

### Clinical characteristics of patients with CRC in TCGA cohort and FUSCC cohort

In the TCGA cohort, the median age of all 356 CRC patients was 66, ranging from 31 to 90 years old. 199 (55.9 %) were male patients and 157 (44.1 %) were female patients. The median follow-up time was 13.4 months and 68 patients died during follow-up. In the FUSCC cohort, the median age of 276 CRC patients was 57, ranging from 27 to 85 years old. 166 (60.1 %) were male and 110 (39.9 %) were female. The median follow-up time was 61.0 months and 81 patients died during follow-up. Tumor primary site, histological type, TNM stage, pathological grading, venous/nervous invasion, extent of TILs, MS/MMR status, pretreatment CEA level and adjuvant therapy in TCGA and FUSCC cohorts are shown in Table 1. In the cohort of TCGA, PD-1 expression was associated with primary site, TILs extent and MS status (*P* < 0.05), whereas PD-L1 expression was correlated with venous invasion, TILs extent, MS status and pretreatment CEA level (*P* < 0.05). In the FUSCC cohort, PD-1 expression in TILs was only associated with M stage (*P* < 0.05), whereas PD-L1 expression in TCs was correlated with T stage and M stage (*P* < 0.05).

**Table 1:** Comparison of baseline clinicopathological characteristics based on PD-1 and PD-L1 expression of CRC patients in TCGA and FUSCC cohorts

	TCGA Cohort (*N* = 356)							FUSCC Cohort (*N* = 276)						
	Cases	PD-1		*P* ^*a*^	PD-L1		*P* ^*a*^	Cases	PD-1		*P* ^*a*^	PD-L1		*P* ^*a*^
	No. (%)	Low	High		Low	High		No. (%)	Low	High		Low	High	
Age (years)														
≤ 60	137 (38.5)	68 (41.2)	69 (36.1)	0.325	21 (38.2)	116 (38.5)	0.960	170 (61.6)	100 (58.8)	70 (66.0)	0.231	85 (61.6)	85 (61.6)	1.000
> 60	219 (35.7)	97 (58.8)	122 (63.9)		34 (61.8)	185 (61.5)		106 (8.4)	70 (41.7)	36 (34.0)		53 (38.4)	53 (38.4)	
Gender														
Male	199 (55.9)	91 (55.2)	108 (56.5)	0.792	33 (60.0)	166 (55.1)	0.505	166 (60.1)	102 (60.0)	64 (60.4)	0.950	88 (63.8)	78 (56.5)	0.219
Female	157 (44.1)	74 (44.8)	83 (43.5)		22 (40.0)	135 (44.9)		110 (39.9)	68 (40.0)	42 (39.6)		50 (36.2)	60 (43.5)	
Primary site														
Colon	269 (75.6)	115 (69.7)	154 (80.6)	*0.017*	46 (83.6)	223 (74.1)	0.130	129 (46.7)	84 (49.4)	45 (42.5)	0.260	65 (47.1)	64 (46.4)	0.904
Rectum	87 (24.4)	50 (30.3)	37 (19.4)		9 (16.4)	78 (25.9)		147 (53.3)	86 (50.6)	61 (57.5)		73 (52.9)	74 (53.6)	
Histological type														
Adenocarcinoma	316 (88.8)	149 (90.3)	167 (87.4)	0.393	50 (90.9)	266 (88.4)	0.584	261 (94.6)	158 (92.9)	103 (97.2)	0.132	132 (95.6)	129 (93.5)	0.426
Mucinous/SRCC	40 (11.2)	16 (9.7)	24 (12.6)		5 (9.1)	35 (11.6)		15 (5.4)	12 (7.1)	3 (2.8)		6 (4.3)	9 (6.5)	
T stage														
Tis-T2	68 (19.1)	29 (17.6)	39 (20.4)	0.386	9 (16.4)	78 (25.9)	0.844	43 (15.6)	24 (14.1)	19 (17.9)	0.588	19 (13.8)	24 (17.4)	*0.044*
T3	242 (68.0)	118 (71.5)	124 (64.9)		39 (70.9)	203 (67.4)		32 (19.6)	32 (18.8)	22 (20.8)		20 (14.5)	34 (24.6)	
T4	46 (12.9)	18 (10.9)	28 (14.7)		7 (12.7)	39 (13.0)		179 (64.8)	114 (67.1)	65 (61.3)		99 (71.7)	80 (58.0)	
N stage														
N0	194 (54.5)	83 (50.3)	111 (58.1)	0.185	23 (41.8)	171 (56.8)	0.101	120 (43.5)	70 (41.2)	50 (47.2)	0.432	57 (41.3)	63 (45.7)	0.767
N1	98 (27.5)	53 (32.1)	45 (23.6)		18 (32.7)	80 (26.6)		83 (30.1)	49 (28.8)	33 (31.1)		43 (31.2)	40 (29.0)	
N2	64 (18.0)	29 (17.6)	35 (18.3)		14 (25.5)	50 (16.6)		73 (26.4)	50 (29.4)	23 (21.7)		38 (27.5)	35 (25.4)	
M stage														
M0	245 (68.8)	109 (66.1)	136 (71.2)	0.107	34 (61.8)	211 (70.1)	0.284	234(84.8)	137 (80.6)	97 (91.5)	*0.01*	108(78.3)	126 (91.3)	*0.003*
M1	48 (13.5)	29 (17.6)	19 (9.9)		11 (20.0)	37 (12.3)		42(15.2)	33 (19.4)	9 (8.5)		30(21.7)	12 (8.7)	
Mx	63 (17.7)	27 (16.4)	36 (18.8)		10 (18.2)	53 (17.6)								
Pathological grading														
Well/moderate	NA							204(73.9)	67 (73.6)	137 (74.1)	0.498	102(73.9)	102 (73.9)	1.000
Poor/anaplastic								56(20.3)	18 (19.8)	38 (20.5)		28(20.3)	28 (20.3)	
Unknown								16(5.8)	6 (6.6)	10 (5.4)		8(5.8)	8 (5.8)	
Venous invasion														
Negative	235 (66.0)	105 (63.6)	130 (68.1)	0.557	28 (50.9)	207 (68.8)	*0.026*	184(66.7)	107 (62.9)	77 (72.6)	0.104	85(61.6)	99 (71.7)	0.193
Positive	70 (19.7)	33 (20.0)	37 (19.4)		14 (25.5)	56 (18.6)		88(31.9)	59 (34.7)	29 (27.4)		51(37.0)	37 (26.8)	
Unknown	51 (14.3)	27 (16.4)	24 (12.6)		13 (23.6)	38 (12.6)		4(1.4)	4 (2.4)	0 (0.0)		2(1.4)	2 (1.4)	
Nervous invasion														
Negative	NA							231 (83.7)	75 (82.4)	156 (84.3)	0.181	118 (85.5)	231 (83.7)	0.337
Positive								45 (16.3)	16 (17.6)	29 (15.7)		20 (14.5)	45 (16.3)	
TILs extent														
Low	47 (13.2)	33 (20.0)	14 (7.3)	*0.002*	16 (29.1)	31 (10.3)	*<0.001*	129 (46.7)	85 (50.0)	44 (41.5)	0.169	59 (42.8)	70 (50.7)	0.184
High	93 (26.1)	42 (25.5)	51 (26.3)		17 (30.9)	76 (25.2)		147 (53.3)	85 (50.0)	62 (58.5)		79 (57.2)	68 (49.3)	
Unknown	216 (60.7)	90 (54.5)	126 (66.0)		22 (40.0)	194 (64.5)								
No. of LNs dissected														
< 12	80 (22.5)	42 (25.5)	38 (19.9)	0.219	14 (25.5)	56 (18.6)	0.658	39 (14.1)	24 (14.1)	15 (14.2)	0.994	16 (11.6)	23 (16.7)	0.226
≥ 12	247 (69.4)	107(64.8)	140 (73.3)		38 (69.1)	209 (69.4)		237 (85.9)	146 (85.9)	91 (85.8)		122 (88.4)	115 (83.3)	
Unknown	29 (8.1)	16 (9.7)	13 (6.8)		3 (5.5)	26 (8.6)								
MS status/MMR status														
MSS/MMR-proficient	243 (68.3)	123 (74.5)	120 (62.8)	*0.018*	39 (70.9)	204 (67.8)	0.646	176 (63.8)	102 (60.0)	74 (69.8)	0.099	93 (67.4)	83 (60.1)	0.21
MSI/MMR-deficient	113 (31.7)	42 (25.5)	71 (37.2)		16 (29.1)	97 (32.2)		100 (36.2)	68 (40.0)	32 (30.2)		45 (32.6)	55 (39.9)	
CEA (μl/ml)														
≤ 5	144 (40.4)	59 (35.8)	85 (44.5)	0.151	13 (23.6)	131 (43.5)	*0.007*	170 (61.6)	106 (62.4)	64 (60.4)	0.532	80 (58.0)	90 (65.2)	0.382
> 5	85 (23.9)	39 (23.6)	46 (24.1)		13 (23.6)	72 (23.9)		95 (34.4)	59 (34.7)	36 (34.0)		51 (37.0)	44 (31.9)	
Unknown	127 (35.7)	67 (40.6)	60 (31.4)		29 (52.7)	98 (32.6)		11 (4.0)	5 (2.9)	6 (5.7)		7 (5.1)	4 (2.9)	
Adjuvant chemotherapy														
Yes	NA							189 (68.5)	58 (63.7)	131 (70.8)	0.082	89 (64.5)	100 (72.5)	0.05
No								49 (17.8)	15 (16.5)	34 (18.4)		23 (16.7)	26 (18.8)	
Unknown								38 (13.7)	18 (19.8)	20 (10.8)		26 (18.8)	12 (8.7)	

^a^Italic entries indicate statistical significance

### Expression pattern of PD-1 and PD-L1

**Fig. 1:**
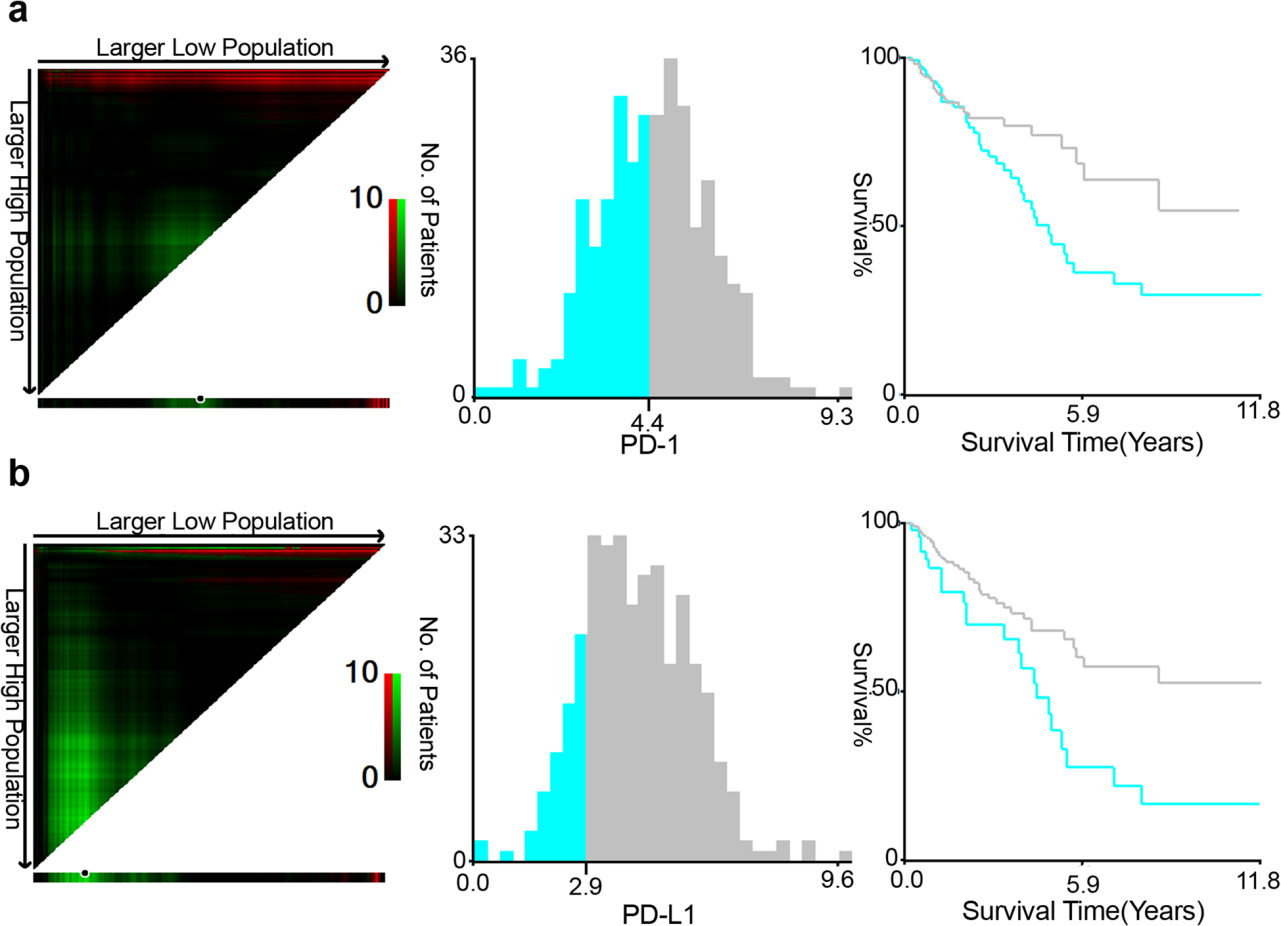
Determination of cut-off values of PD-1 and PD-L1 expressions in TCGA database and survival analyses. X-tile analysis of 5-year OS was performed using patients’ data in TCGA database to determine the optimal cut-off value for PD-1 and PD-L1 expression. The sample of CRC patients was equally divided into training and validation sets. X-tile plots of training sets are shown in the *left panels*, with plots of matched validation sets shown in the *smaller inset*. The optimal cut-off values highlighted by the *black circles* in *left panels* are shown in histograms of the entire cohort (*middle panels*), and Kaplan-Meier plots are displayed in *right panels. P* values were determined by using the cut-off values defined in training sets and applying them to validation sets. **a** The optimal cut-off value for PD-1 was 4.40 (χ^2^ = 4.591, *P* = 0.032). **b** The optimal cut-off value for PD-L1 was 2.92 (χ^2^ = 9.349, *P* = 0.002)

In the TCGA cohort, the cut-off values of PD-1 and PD-L1 mRNA levels in tumor tissue of eligible patients were determined by X-tile program, which were 4.40 and 2.92, respectively (Fig. 1). The χ^2^ log-rank value of PD-1 and PD-L1 were 4.591 and 9.349, respectively. Patients were divided into 2 groups for further analysis (PD-1 ≤ 4.40 and >4.40, PD-L1 ≤ 2.92 and >2.92).

**Fig. 2:**
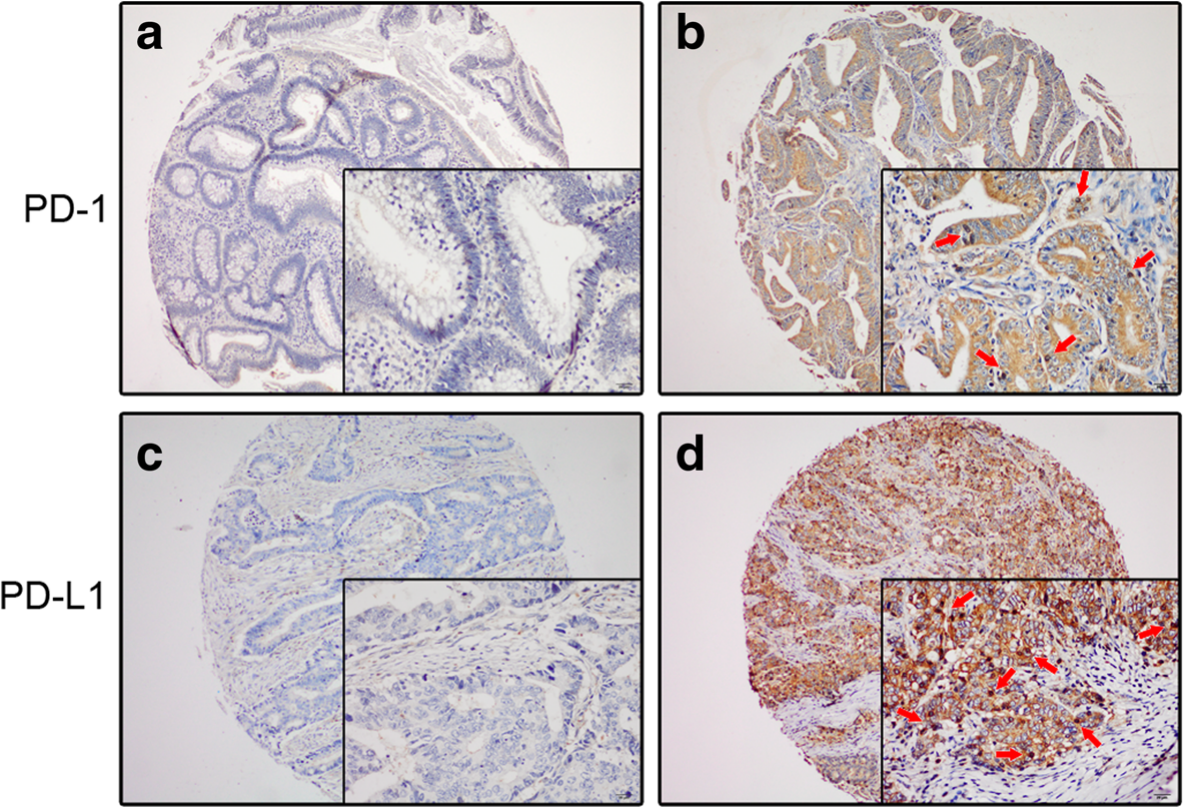
IHC staining of PD-L1 and PD-1 expressions in TMA samples. PD-1 displayed a membrane-accentuated expression, which was often accompanied by a cytoplasmic expression (**b**), while PD-L1showed a membrane-accentuated expression (**d**). Tumor infiltrating lymphocytes were indicated by *red arrows*. The representative pictures of negative expression of PD-1/PD-L1 were also shown (**a**, **c**)

In the FUSCC cohort, the expressions of PD-1 in TILs and PD-L1 in tumor cells (TCs) were detected by IHC staining. PD-L1 showed a membrane-accentuated expression, while PD-1 also displayed a membrane-accentuated expression, which was often accompanied by a cytoplasmic expression (Fig. 2). For further analysis, patients were divided into two groups with low expression of PD-1/PD-L1 (IRS ≤ 4) and high expression of them (IRS > 4). In 106 CRC patients (38.4 %), a high expression of PD-1 in TILs was observed and in 170 cases (61.6 %), PD-1 expression in TILs was low. While half of the cases in this cohort displayed a high expression of PD-L1 in TCs, and the other half showed a low expression of it.

### Prognostic significance of PD-1 and PD-L1 expression

In the TCGA cohort, the univariate Cox regression model revealed that TNM stages, venous invasion, pretreatment CEA level, PD-1 and PD-L1 expressions were associated with prognosis of CRC patients in terms of OS (*P* < 0.05, Table 2). Multivariate analysis after adjustment indicated that only T stage and pretreatment CEA level were independent prognostic factors for OS in CRC patients (*P* < 0.05) and PD-1 and PD-L1 expression lost their significance (*P* > 0.05).

**Table 2:** Univariate and multivariate Cox proportional hazards analysis of OS for patients with CRC in the TCGA cohort

Variables^a^	Univariate analysis HR (95 % CI)	*P* ^*b*^	Multivariate analysis HR (95 % CI)	*P* ^*b*^
Age (years)				
≤ 60	1.000	0.202	1.000	0.937
> 60	1.366 (0.846–2.204)		1.021 (0.606–1.720)	
Gender				
Male	1.000	0.132	1.000	0.205
Female	0.687 (0.421–1.120)		0.718 (0.431–1.198)	
Tumor location				
Colon	1.000	0.149		
Rectum	0.580 (0.277–1.215)			
Histological type				
Adenocarcinoma	1.000	0.148		
Mucinous/SRCC	1.611 (0.844–3.076)			
T stage				
Tis-T2	1.000	*<0.001*	1.000	*<0.001*
T3	1.463 (0.618–3.465)		0.832 (0.330–2.053)	
T4	8.584 (3.316–22.222)		2.912 (1.242–8.093)	
N stage				
N0	1.000	*<0.001*	1.000	0.583
N1	2.311 (1.306–4.091)		1.430 (0.724–2.823)	
N2	3.098 (1.718–5.588)		1.213 (0.557–2.642)	
M stage				
M0	1.000	*<0.001*	1.000	0.115
M1	4.013 (2.252–7.152)		2.062 (1.007–4.221)	
Mx	1.996 (1.083–3.676)		1.701 (0.837–3.458)	
Venous invasion				
Negative	1.000	*0.006*	1.000	0.145
Positive	2.411 (1.400–4.151)		2.008 (1.000–4.033)	
Unknown	1.625 (0.842–3.137)		1.362 (0.655–2.830)	
TILs extent				
Low	1.000	0.541		
High	0.746 (0.357–1.558)			
Unknown	0.737 (0.422–1.286)			
No. of LNs dissected				
< 12	1.000	0.145		
≥ 12	2.311 (0.542–9.853)			
Unknown	1.432 (0.347–5.917)			
MS status				
MSS	1.000			
MSI	1.159 (0.710–1.892)	0.557		
CEA (μl/ml)				
≤ 5	1.000		1.000	*0.011*
> 5	2.146 (0.978–4.708)	*0.003*	1.339 (0.582–3.081)	
Unknown	3.086 (1.617–5.891)		2.778 (1.337–5.772)	
PD-1				
Low	1.000		1.000	0.68
High	0.591 (0.364–0.961)	*0.034*	0.883 (0.488–1.596)	
PD-L1				
Low	1.000		1.000	0.148
High	0.463 (0.279–0.768)	*0.003*	0.626 (0.332–1.181)	

^a^All variables are djusted by Cox proportional hazards models including age, gender, T stage, N stage and M stage

^b^Italic entries indicate statistical significance

**Fig. 3:**
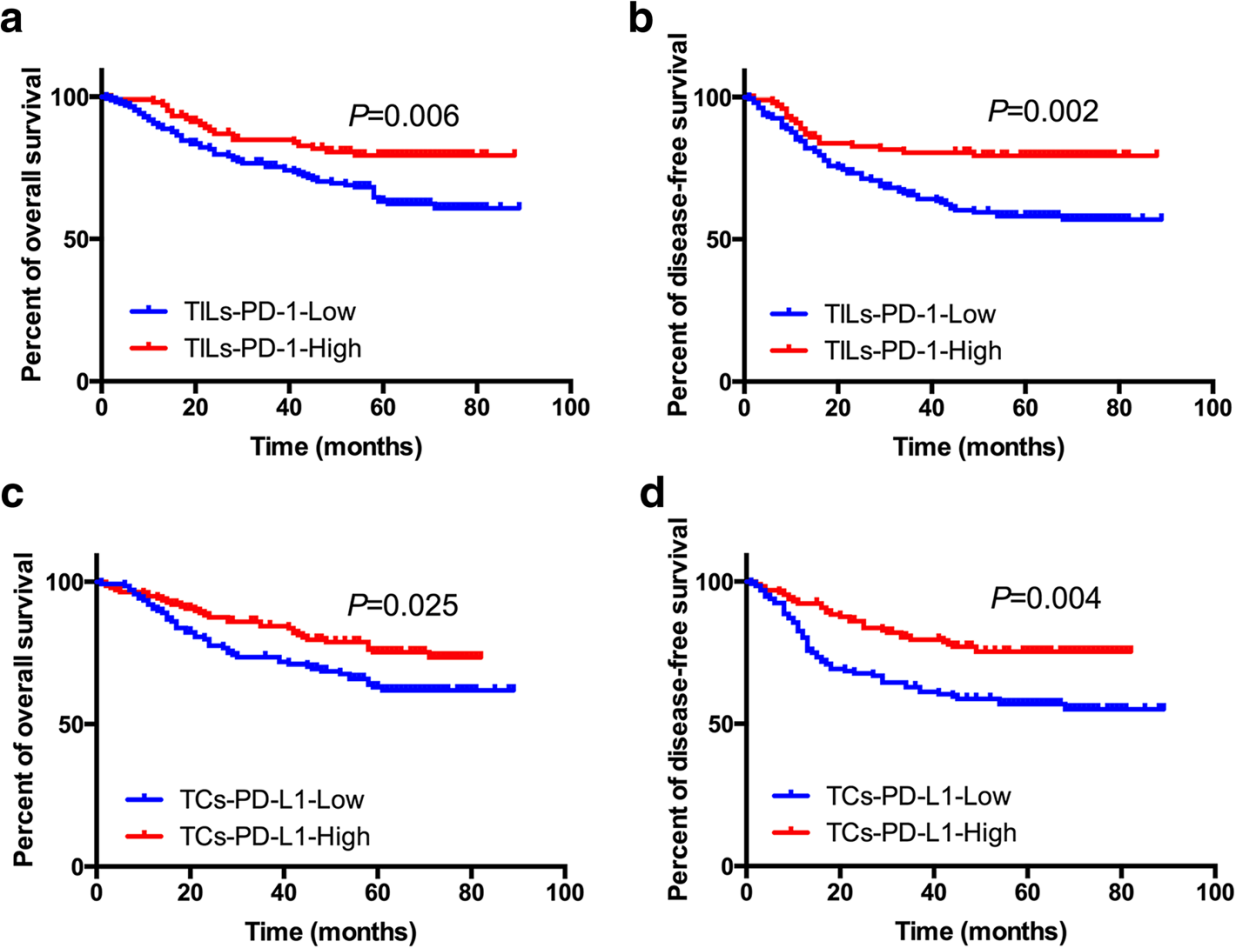
Kaplan Meier analyses for TILs-PD-1/TCs-PD-L1 expression and their correlation with clinical outcome in FUSCC cohort. **a** OS according to TILs-PD-1; **b** DFS according to TILs-PD-1; **c** OS according to TCs-PD-L1; **d** DFS according to TCs-PD-L1

In the FUSCC cohort, Kaplan-Meier analysis showed that both the expression of PD-1 in TILs and the expression of PD-L1 in TCs were associated with OS and DFS of CRC patients (*P* < 0.05, Fig. 3a–d). The univariate Cox regression model indicated that TNM stages, venous invasion, pretreatment CEA level, adjuvant chemotherapy, TILs-PD-1 and TCs-PD-L1 expressions were correlated with OS (*P* < 0.05), whereas TNM stages, venous invasion, nervous invasion, pretreatment CEA level, adjuvant chemotherapy, TILs-PD-1 and TCs-PD-L1 expressions were associated with DFS. Multivariate analysis after adjustment revealed that T stage, M stage, pretreatment CEA level, adjuvant chemotherapy and TILs-PD-1 were independent prognostic factors for OS, while only T stage, M stage and TILs-PD-1 were independent prognostic factor for DFS of CRC patients (Table 3).

**Table 3:** Univariate and multivariate Cox proportional hazards analysis of OS and DFS for patients with CRC in the FUSCC cohort

Variables^a^	OS				DFS			
	Univariate analysis	*P* ^*b*^	Multivariate analysis	*P* ^*b*^	Univariate analysis	*P* ^*b*^	Multivariate analysis	*P* ^*b*^
	HR (95 % CI)		HR (95 % CI)		HR (95 % CI)		HR (95 % CI)	
Age (years)								
≤ 60	1.000	0.367	1.000	0.564	1.000	0.593	1.000	0.633
> 60	1.224 (0.789–1.900)		1.154 (0.709–1.879)		1.115 (0.749–1.661)		1.113 (0.717–1.730)	
Gender^b^								
Male	1.000	0.418	1.000	0.493	1.000	0.238	1.000	0.305
Female	0.829 (0.526–1.306)		0.843 (0.518–1.374)		0.781 (0.518–1.178)		0.793 (0.509–1.235)	
Tumor location								
Colon	1.000	0.061			1.000	0.223		
Rectum	0.658 (0.424–1.020)				0.784 (0.529–1.160)			
Histological type								
Adenocarcinoma	1.000	0.781			1.000	0.765		
Mucinous/SRCC	0.867 (0.317–2.369)				0.872 (0.355–2.143)			
T stage								
Tis-T2	1.000	*<0.001*	1.000	*0.017*	1.000	*<0.001*	1.000	*0.032*
T3	4.450 (0.975–20.312)		6.329 (1.325–30.238)		2.979 (0.971–9.138)		3.471 (1.074–11.221)	
T4	10.534 (2.581–42.992)		8.734 (1.948–39.161)		6.524 (2.390–17.805)		4.282 (1.443–12.710)	
N stage								
N0	1.000	*<0.001*	1.000	0.269	1.000	*<0.001*	1.000	0.089
N1	2.770 (1.537–4.992)		2.011 (0.982–4.117)		2.927 (1.721–4.979)		2.251 (1.202–4.215)	
N2	4.146 (2.336–7.357)		1.931 (0.903–4.130)		4.212 (2.510–7.069)		1.968 (0.984–3.934)	
M stage								
M0	1.000	*<0.001*	1.000	*0.024*	1.000	*<0.001*	1.000	*<0.001*
M1	9.094 (5.757–14.367)		2.833 (1.137–6.083)		10.198 (6.648–15.642)		4.377 (1.999–9.585)	
Pathological grading								
Well/moderate	1.000	0.378			1.000	0.729		
Poor/anaplastic	1.278 (0.762–2.144)				1.137 (0.706–1.832)			
Unknown	0.576 (0.180–1.836)				0.784 (0.317–1.941)			
Venous invasion								
Negative	1.000	*<0.001*	1.000	0.987	1.000	*<0.001*	1.000	0.55
Positive	2.448 (1.578–3.796)		0.986 (0.578–1.681)		2.216 (1.492–3.291)		0.760 (0.464–1.246)	
Unknown	1.043 (0.143–7.587)		1.163 (0.154–8.756)		0.813 (0.112–5.884)		0.821 (0.110–6.136)	
Nervous invasion								
Negative	1.000	0.059			1.000	*0.008*	1.000	0.199
Positive	1.641 (0.981–2.743)				1.186 (1.175–2.949)		1.375 (0.846–2.237)	
No. of LNs dissected								
< 12	1.000	0.249			1.000	0.297		
≥ 12	0.719 (0.410–1.260)				0.757 (0.449–1.276)			
MMR status								
MMR-proficient	1.000	0.726			1.000	0.792		
MMR-deficient	1.084 (0.692–1.696)				0.947 (0.632–1.418)			
CEA (μl/ml)								
≤ 5	1.000	*<0.001*	1.000	*0.041*	1.000	*<0.001*	1.000	0.096
> 5	2.468 (1.572–3.875)		1.386 (0.824–2.331)		2.768 (1.845–4.152)		1.529 (0.956–2.444)	
Unknown	2.850 (1.118–7.265)		3.513 (1.292–9.558)		2.472 (0.979–6.246)		2.230 (0.848–5.863)	
Adjuvant chemotherapy								
Yes	1.000	*<0.001*	1.000	*0.019*	1.000	*<0.001*	1.000	0.366
No	0.670 (0.315–1.425)		1.835 (0.735–4.584)		0.568 (0.281–1.146)		1.419 (0.609–3.307)	
Unknown	8.344 (5.142–13.542)		3.130 (1.345–7.282)		6.990 (4.495–10.870)		1.645 (0.755–3.584)	
TILs-PD-1								
Low	1.000	*0.007*	1.000	*0.006*	1.000	*0.003*	1.000	*0.015*
High	0.500 (0.302–0.828)		0.480 (0.283–0.814)		0.502 (0.319–0.789)		0.559 (0.350–0.893)	
TCs-PD-L1								
Low	1.000	*0.027*	1.000	0.852	1.000	*0.005*	1.000	0.66
High	0.606 (0.389–0.944)		1.048 (0.639–1.719)		0.558 (0.373–0.835)		0.904 (0.577–1.417)	

^a^All variables are djusted by Cox proportional hazards models including age, gender, T stage, N stage and M stage

^b^Italic entries indicate statistical significance

### Subgroup analyses of TILs-PD-1 and TCs-PD-L1 expression according to MMR status in FUSCC cohort

**Fig. 4:**
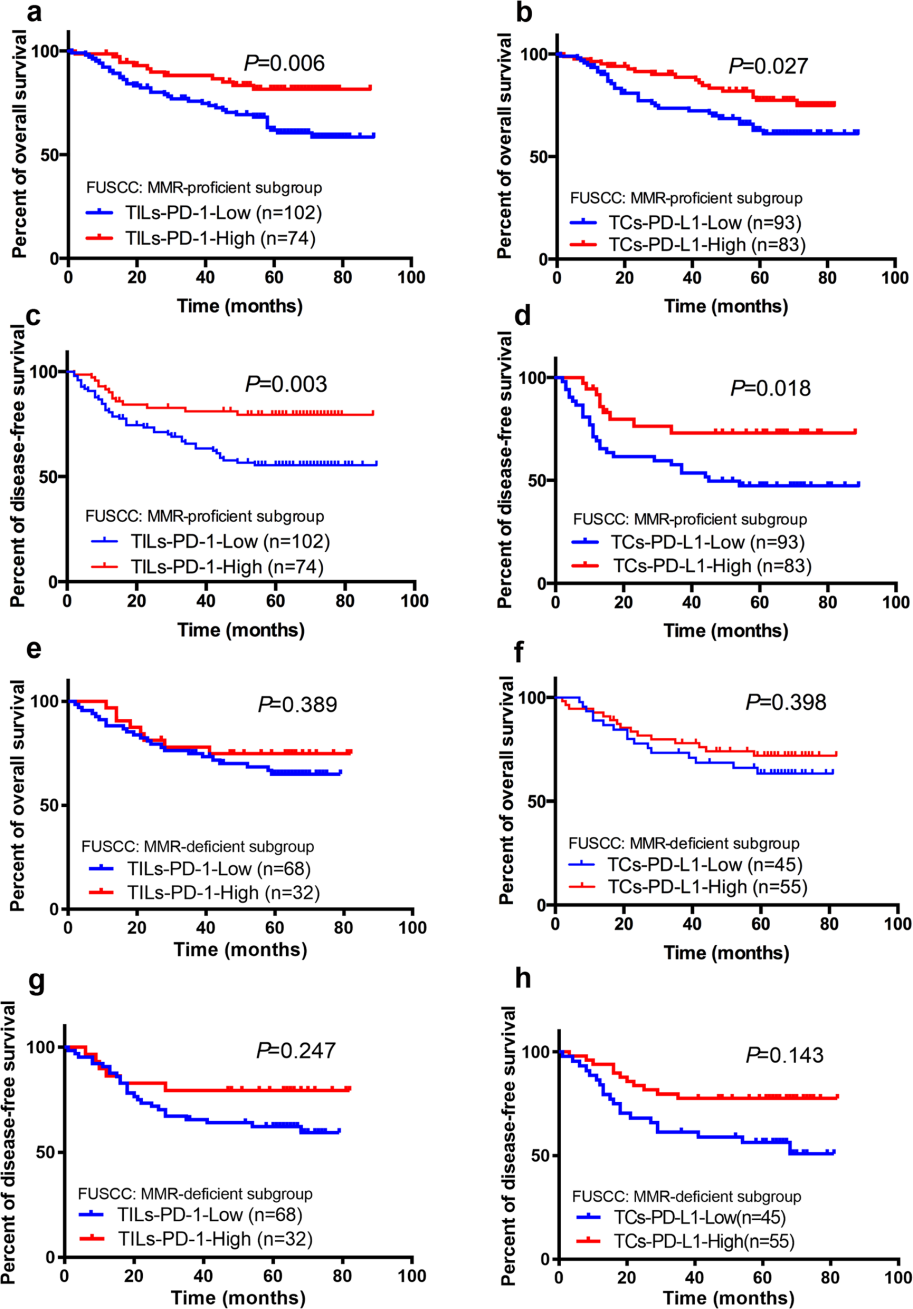
Subgroup analyses using Kaplan Meier method for TILs-PD-1/TCs-PD-L1 expression and their correlation with clinical outcome in FUSCC cohort. **a** OS according to TILs-PD-1 in MMR-proficient subgroup; **b** OS according to TCs-PD-L1 in MMR-proficient subgroup; **c** DFS according to TILs-PD-1 in MMR-proficient subgroup; **d** DFS according to TCs-PD-L1 in MMR-proficient subgroup; **e** OS according to TILs-PD-1 in MMR-deficient subgroup; **f** OS according to TCs-PD-L1 in MMR-deficient subgroup; **g** DFS according to TILs-PD-1 in MMR-deficient subgroup; **h** DFS according to TCs-PD-L1 in MMR-deficient subgroup

Previous studies indicated that active immune microenvironment was observed in MSI/MMR-deficient tumors [19], which may lead to a better prognosis of CRC patients. And it was reported that only microsatellite instable (MSI) subset of CRC seems to be a good candidate for checkpoint blockage immunotherapy [3] and the mechanisms are still controversial. Thus, we further made subgroup analyses of PD-1 and PD-L1 expressions according to patients’ MMR status in FUSCC cohort. Kaplan-Meier analysis indicated that both TILs-PD-1 and TCs-PD-L1 correlated with OS and DFS (*P* < 0.05, Fig. 4a–d) in MSS-proficient subgroup while neither of TILs-PD-1 and TCs-PD-L1 correlated with OS or DFS in MSS-deficient subgroup (*P* > 0.05, Fig. 4e–h). In the MSS-proficient subgroup, the univariate Cox regression model revealed that TNM stages, adjuvant chemotherapy, TILs-PD-1 and TCs-PD-L1 expressions were associated with prognosis of CRC patients in terms of OS (*P* < 0.05, Additional file 1: Table S1), whereas TNM stages, venous and nervous invasion, pretreatment CEA level, adjuvant chemotherapy, TILs-PD-1 and TCs-PD-L1 expressions were associated with prognosis of CRC patients in terms of DFS (*P* < 0.05). However, multivariate analysis after adjustment indicated that only TILs-PD-1 was an independent prognostic factor for OS and only M stage and TILs-PD-1 were independent prognostic factors for DFS in CRC patients (*P* < 0.05). In the MSS-deficient subgroup, the Cox regression model revealed that neither of TILs-PD-1 and TCs-PD-L1 was an independent prognostic factor for OS or DFS in CRC patients (*P* > 0.05, Additional file 2: Table S2).

## Discussion

PD-1/PD-L1 axis, as an immune checkpoint, is usually up-regulated to create an immunosuppressive tumor microenvironment and help cancer cells escape immune-mediated destruction [20]. Previous studies indicated that the correlations between PD-L1 and prognosis are variant among different tumor types [21–24]. For colorectal cancer, Droeser RA et al. found that PD-L1 expression is paradoxically associated with improved survival in MMR-proficient CRC [25]. However, the expression of PD-1 was not analyzed in their study. Our study aimed at systematically analyzing the expression of PD-1 and PD-L1 in colorectal cancer cells and tumor infiltrating cells, and their clinical significance, adopting TCGA database and the FUSCC cohort of patients. We found that the high expression of PD-1 or PD-L1 were associated with better prognosis of CRC patients and PD-1 expression in TILs was an independent prognostic factor for OS and DFS of CRC patients, specifically for patients in MMR-proficient status.

We firstly used TCGA database to investigate the relationship between the expression of PD-1/PD-L1 and clinical outcome. To begin with, X-tile program, a robust graphic tool, was adopted to identify the optimal cut-off values of PD-1 and PD-L1 levels. The Kaplan-Meier analysis revealed the positive correlation of higher PD-1/PD-L1 expression and better OS of CRC patients, lying the basis of our study. However, the PD-1/PD-L1 level in TCGA database was detected by RNA sequencing using the whole RNA extracted in tumor tissue quantifying expression level of both TCs and TILs. In fact, PD-1 and PD-L1 are expressed in two tumoral compartments, tumor cells and TILs respectively, and their interaction may cause changes in tumor microenvironment, leading to different results of tumor progression. Thus, TILs-PD-1 and TCs-PD-L1 were then analyzed separately by IHC detection based on a relatively large TMA sample in FUSCC to verify the results in TCGA database. Consistently, both of the biomarkers were associated with better OS and DFS of CRC patients.

Currently, the expression of PD-L1 on TCs is regarded as an immune-tolerance mechanism of the tumor, as it can attract PD-1 expressing immune-inhibitory TILs. However, this mechanism is expected to result into a negative correlation of TILs-PD-1/TCs-PD-L1 expression and survival, as it was reported, e.g. for renal cell carcinoma, breast cancer, non-small cell lung cancer [24] and osteosarcoma [26]. Of note, the present study is not the only one to report a favorable prognostic impact of TILs-PD-1/TCs-PD-L1 expression in cancer cells. Sabatier et al. [22] investigated the *PDL1* level in 5, 454 breast cancers using DNA microarray and demonstrated that higher *PDL1* expression was associated with better metastasis-free survival and OSS and better response to chemotherapy. Additionally, Darb-Esfahani et al. [27] detected the PD-1 and PD-L1 protein expression by IHC on TMA from 215 primary ovary cancers and described that PD-1 and PD-L1 expression in TCs were positive prognostic factors for progression-free survival and OS.

Now, it can only be speculated why the up-regulation of PD-1/PD-L1 axis are in some instances (such as in CRC in the present study) referred to a favorable clinical outcome. A positive impact of PD-L1 expression of TCs might be explained by a compensatory up-regulation of this marker in a microenvironment that threatens the tumor by an active immune response. An association between TCs-PD-L1 and a high TILs density would be an argument for this hypothesis, and has been well illustrated in breast cancer [22, 28]. However this hypothesis remains speculative. The positive prognostic impact of TILs-PD-1 may also base on regulatory and not yet completely elucidated mechanisms within the immune network in the tumor microenvironment. Therefore, regulatory and immune-suppressive T cells might be up-regulated during an enhanced anti-tumoral immune response.

Previous studies indicated that active immune microenvironment was observed in MSI/MMR-deficient tumors [19], and these tumors are characterized by a more favorable prognosis compared to MSS/MMR-proficient tumors. MSI is typically diagnosed by the variable lengths of DNA microsatellites (mononucleotide and dinucleotide repeats), which is caused by epigenetic silencing or mutation of DNA MMR genes, leading to accumulated mutations at 10–100 times the normal rate and promotes MSI carcinoma progression [19]. In the MSI/MMR-deficient subset of CRC, the high accumulated mutation creates many tumor-specific neoantigens, typically 10–50 times that of MSS/MMR-proficient subset [29], which might be the reason for the high level of TILs and active Th1/CTL immune microenvironment in MSI/MMR-tumors observed in many previous studies [19]. In the present study, subgroup analyses were carried out in FUSCC cohort and demonstrated that higher expressions of TILs-PD-1 and TCs-PD-L1 were associated with better prognosis in MMR-proficient subgroup while no correlation was found between TILs-PD-1/TCs-PD-L1 expression and prognosis. Surprisingly, TILs-PD-1 was even an independent factor for OS and DFS of CRC patients. The underline mechanisms remain unclear. IFN-γ, mainly produced by activated infiltrating T cells, has been reported to be associated with a favorable prognosis [30] and has been shown to promote the expression of PD-1 ligands in different cell types, which might mirror the co-overexpression of TILs-PD-1 and TCs-PD-L1 in MMR-proficient CRC. In the present study, among 356 eligible patients included in TCGA database, 250 patients have the data of IFN-γ gene expression. Spearman test showed that both PD-1 and PDL-1 are highly directly correlated with IFN-γ with *r* = 0.7285 (*P* < 0.0001, 95 % CI: 0.6624–0.7833) and *r* = 0.7475 (*P* < 0.0001, 95 % CI: 0.6853–0.7990), which supported the above hypothesis (Additional file 3: Figure S1). On the other hand, the intestinal immune system is shaped by a continuous interaction with commensal microbiota [31]. Possibly, as a consequence of this specific microenvironment, CRC infiltration by immunocompetent cells is associated with paradoxically peculiar features [32]. Indeed, previous studies [33] demonstrated that, in contrast to a wide range of human cancers, CRC infiltration by FOXP3+ regulatory T cells, is associated with an improved prognosis. Furthermore, it has also been observed that CRC infiltration by myeloid cells is also associated with a favorable prognosis [34].

To our knowledge, the present study is the first study based on East Asia patients concerning PD-1 and PD-L1 analyses, which combines TCGA database and center-based cohort to comprehensively and specifically investigate TILs-PD-1 and TCs-PD-L1 and their clinical relevance in CRC. However, our study has several limitations. First, the insufficient data on recurrence in CRC patients from in TCGA database limited the analyses on the impact of PD-1/PD-L1 expression for DFS. And the TCGA database does not include information regarding the pathological grading, nervous invasion and administration of chemotherapy and all these factors may affect the multivariate analysis of TCGA data. Secondly, although we have analyzed a large cohort, the present study is a retrospective analysis and there is a potential for selection bias. A relatively small sample of the MMR-deficient subgroup may result in lack of power for the Cox regression analysis and some of potential correlation between TILs-PD-1/TCs-PD-L1 expression and clinical outcome may fail to manifest. Thirdly, it is difficult to compare the results from our study with previous studies due to different antibodies and variant methodologies used to evaluate TILs-PD-1/TCs-PD-L1 expression.

## Conclusion

In conclusion, higher expression of PD-1 and PD-L1 is associated with better prognosis of CRC patients in TCGA and FUSCC cohort. TILs-PD-1 expression was an independent prognostic factor for OS and DFS of CRC patients, especially for MMR-proficient tumors. Future investigations should focus on PD-1/PD-L1 expression on TILs, instead of only on TCs.

## Additional files

Additional file 1: Table S1. Univariate and multivariate Cox proportional hazards analysis of OS and DFS for patients with CRC in MMR-proficient subgroup of the FUSCC cohort. (DOCX 18 kb) Additional file 2: Table S2. Univariate and multivariate Cox proportional hazards analysis of OS and DFS for patients with CRC in MMR-deficient subgroup of the FUSCC cohort. (DOCX 17 kb) Additional file 3: Figure S1. Correlation of PD-1/PD-L1 and IFN-γ in TCGA database. In TCGA cohort, 250 patients have the data of IFN-γ gene expression. Spearman test was used to determine the correlation of PD-1/PD-L1 and IFN-γ. (a) The expression of PD-1 is directly correlated with IFN-γ (*P* < 0.0001, *r* = 0.7285, 95 % CI: 0.6624–0.7833). (b) The expression of PD-L1 is directly correlated with IFN-γ (*P* < 0.0001, *r* = 0.7475, 95 % CI: 0.6853–0.7990). (TIF 1066 kb)

## Abbreviations

CRC: Colorectal cancer
FUSCC: Fudan University Shanghai Cancer Center
IRS: Immunoreactivity score
MMR: Mismatch repair
MS: Microsatellite
SRCC: Signet ring colorectal cancer
TCGA: The cancer genome atlas
TCs: Tumor cells
TILs: Tumor infiltrating cells
