# Supplementary material for: Prognostic impact of programed cell death-1 (PD-1) and PD-ligand 1 (PD-L1) expression in cancer cells and tumor infiltrating lymphocytes in colorectal cancer
Source: Mol Cancer. 2016 Aug 24;15(1):55. doi: 10.1186/s12943-016-0539-x (PMC4995750; doi:10.1186/s12943-016-0539-x)
Supplement: Additional file 1: Table S1. — Univariate and multivariate Cox proportional hazards analysis of OS and DFS for patients with CRC in MMR-proficient subgroup of the FUSCC cohort. (DOCX 18 kb) [file 12943_2016_539_MOESM1_ESM.docx]

| **Table S1**. Univariate and multivariate Cox proportional hazards analysis of OS and DFS for patients with CRC in MMR-proficient subgroup of the FUSCC cohort. | | | | | | | | |
| --- | --- | --- | --- | --- | --- | --- | --- | --- |
| Variables ^a^ | OS | | | | DFS | | | |
|  | Univariate analysis | *P ^b^* | Multivariate analysis | *P ^b^* | Univariate analysis | *P ^b^* | Multivariate analysis | *P ^b^* |
|  | HR (95%CI) |  | HR (95%CI) |  | HR (95%CI) |  | HR (95%CI) |  |
| Age (years) | | | | | | | | |
| ≤60 | 1.000 | 0.323 | 1.000 | 0.644 | 1.000 | 0.709 | 1.000 | 1.000 |
| >60 | 1.323 (0.760-2.304) |  | 1.154 (0.629-2.117) |  | 1.100 (0.666-1.818) |  | 1.000 (0.567-1.765) |  |
| Gender | | | | | | | | |
| Male | 1.000 | 0.744 | 1.000 | 0.741 | 1.000 | 0.667 | 1.000 | 0.743 |
| Female | 1.101 (0.618-1.962) |  | 1.110 (0.596-2.069) |  | 1.120 (0.669-1.877) |  | 1.098 (0.629-1.916) |  |
| Tumor location | | | | | | | | |
| Colon | 1.000 | 0.155 |  |  | 1.000 | 0.412 |  |  |
| Rectum | 0.667 (0.383-1.164) |  |  |  | 0.812 (0.493-1.336) |  |  |  |
| Histological type | | | | | | | | |
| Adenocarcinoma | 1.000 | 0.844 |  |  | 1.000 | 0.519 |  |  |
| Mucinous/SRCC | 0.890 (0.277-2.860) |  |  |  | 0.683 (0.214-2.178) |  |  |  |
| T stage | | | | | | | | |
| Tis-T2 | 1.000 | *0.004* | 1.000 | 0.298 | 1.000 | *0.002* | 1.000 | 0.251 |
| T3 | 3.120 (0.662-14.695) |  | 3.243 (0.656-16.026) |  | 3.046 (0.850-10.921) |  | 3.063 (0.808-11.617) |  |
| T4 | 7.281 (1.758-30.153) |  | 3.356 (0.715-15.755) |  | 6.130 (1.907-19.702) |  | 2.606 (0.728-9.326) |  |
| N stage | | | | | | | | |
| N0 | 1.000 | *<0.001* | 1.000 | 0.23 | 1.000 | *<0.001* | 1.000 | 0.066 |
| N1 | 2.494 (1.178-5.281) |  | 1.302 (0.535-3.172) |  | 3.131 (1.567-6.257) |  | 2.212 (0.988-4.953) |  |
| N2 | 4.287 (2.114-8.695) |  | 2.003 (0.865-4.638) |  | 5.076 (2.624-9.819) |  | 2.603 (1.133-5.979) |  |
| M stage | | | | | | | | |
| M0 | 1.000 | *<0.001* | 1.000 | 0.052 | 1.000 | *<0.001* | 1.000 | *0.01* |
| M1 | 8.827 (4.938-15.777) |  | 2.643 (0.992-7.041) |  | 9.132 (5.289-15.769) |  | 3.573 (1.355-9.424) |  |
| Pathological grading | | | | | | | | |
| Well/moderate | 1.000 | 0.527 |  |  | 1.000 | 0.612 |  |  |
| Poor/anaplastic | 1.164 (0.593-2.283) |  |  |  | 1.106 (0.607-2.015) |  |  |  |
| Unknown | 0.487 (0.117-2.023) |  |  |  | 0.589 (0.183-1.895) |  |  |  |
| Venous invasion | | | | | | | | |
| Negative | 1.000 | 0.07 |  |  | 1.000 | *0.024* | 1.000 | 0.766 |
| Positive | 1.930 (1.103-3.376) |  |  |  | 2.008 (1.217-3.314) |  | 0.796 (0.432-1.468) |  |
| Unknown |  |  |  |  |  |  |  |  |
| Nervous invasion | | | | | | | | |
| Negative | 1.000 | 0.242 |  |  | 1.000 | *0.031* | 1.000 | 0.788 |
| Positive | 1.491 (0.763-2.911) |  |  |  | 1.897 (1.058-3.400) |  | 1.095 (0.566-2.117) |  |
| No. of LNs dissected | | | | | | | | |
| <12 | 1.000 | 0.066 |  |  | 1.000 | 0.107 |  |  |
| ≥12 | 0.544 (0.284-1.042) |  |  |  | 0.605 (0.328-1.115) |  |  |  |
| CEA (μl/ml) | | | | | | | | |
| ≤5 | 1.000 | 0.08 |  |  | 1.000 | *0.007* | 1.000 | 0.573 |
| >5 | 1.913 (1.079-3.392) |  |  |  | 2.277 (1.365-3.800) |  | 1.284 (0.726-2.269) |  |
| Unknown | 1.676 (0.505-5.568) |  |  |  | 1.538 (0.467-5.060) |  | 1.643 (0.470-5.742) |  |
| Adjuvant chemotherapy | | | | | | | | |
| Yes | 1.000 | *<0.001* | 1.000 | 0.088 | 1.000 | *<0.001* | 1.000 | 0.371 |
| No | 0.355 (0.108-1.163) |  | 0.720 (0.182-2.845) |  | 0.266 (0.082-0.859) |  | 0.640 (0.166-2.460) |  |
| Unknown | 7.783 (4.172-14.522) |  | 3.225 (1.101-9.446) |  | 6.354 (3.579-11.279) |  | 1.848 (0.692-4.936) |  |
| TILs-PD-1 | | | | | | | | |
| Low | 1.000 | *0.008* | 1.000 | *0.011* | 1.000 | *0.004* | 1.000 | *0.009* |
| High | 0.413 (0.216-0.790) |  | 0.420 (0.216-0.819) |  | 0.435 (0.246-0.768) |  | 0.444 (0.241-0.816) |  |
| TCs-PD-L1 | | | | | | | | |
| Low | 1.000 | *0.03* | 1.000 | 0.973 | 1.000 | *0.014* | 1 | 0.699 |
| High | 0.528 (0.296-0.941) |  | 0.988 (0.500-1.952) |  | 0.519 (0.308-0.873) |  | 0.884 (0.473-1.651) |  |
| a All variables are djusted by Cox proportional hazards models including age, gender, T stage, N stage and M stage. b Italic entries indicate statistical significance. | | | | | | | | |
