# Supplementary material for: Prognostic impact of programed cell death-1 (PD-1) and PD-ligand 1 (PD-L1) expression in cancer cells and tumor infiltrating lymphocytes in colorectal cancer
Source: Mol Cancer. 2016 Aug 24;15(1):55. doi: 10.1186/s12943-016-0539-x (PMC4995750; doi:10.1186/s12943-016-0539-x)
Supplement: Additional file 2: Table S2. — Univariate and multivariate Cox proportional hazards analysis of OS and DFS for patients with CRC in MMR-deficient subgroup of the FUSCC cohort. (DOCX 17 kb) [file 12943_2016_539_MOESM2_ESM.docx]

| **Table S2.** Univariate and multivariate Cox proportional hazards analysis of OS and DFS for patients with CRC in MMR-deficient subgroup of the FUSCC cohort | | | | | | | | |
| --- | --- | --- | --- | --- | --- | --- | --- | --- |
| Variables ^a^ | OS | | | | DFS | | | |
|  | Univariate analysis | *P ^b^* | Multivariate analysis | *P ^b^* | Univariate analysis | *P ^b^* | Multivariate analysis | *P ^b^* |
|  | HR (95%CI) |  | HR (95%CI) |  | HR (95%CI) |  | HR (95%CI) |  |
| Age (years) | | | | | | | | |
| ≤60 | 1.000 | 0.815 | 1.000 | 0.649 | 1.000 | 0.673 | 1.000 | 0.660 |
| >60 | 1.092 (0.523-2.279) |  | 0.824 (0.358-1.898) |  | 1.153 (0.596-2.229) |  | 0.850 (0.412-1.754) |  |
| Gender | | | | | | | | |
| Male | 1.000 | 0.091 | 1.000 | 0.127 | 1.000 | *0.017* | 1.000 | 0.050 |
| Female | 0.530 (0.254-1.107) |  | 0.512 (0.216-1.211) |  | 0.435 (0.219-0.862) |  | 0.452 (0.205-1.000) |  |
| Tumor location | | | | | | | | |
| Colon | 1.000 | 0.251 |  |  | 1.000 | 0.39 |  |  |
| Rectum | 0.658 (0.323-1.344) |  |  |  | 0.755 (0.399-1.432) |  |  |  |
| Histological type | | | | | | | | |
| Adenocarcinoma | 1.000 | 0.843 |  |  | 1.000 | 0.585 |  |  |
| Mucinous/SRCC | 0.818 (0.111-5.997) |  |  |  | 1.487 (0.358-6.186) |  |  |  |
| T stage | | | | | | | | |
| Tis-T2 | 0.000 (0.000-2.876E+267) | 0.44 | 0.000 (0.000-4.576E+228) | 0.791 | 1.000 | *0.041* | 1.000 | *0.033* |
| T3 | 0.392 (0.093-1.644) |  | 0.567 (0.107-2.997) |  | 2.375 (0.215-26.191) |  | 3.059 (0.242-38.674) |  |
| T4 | 1.000 |  | 1.000 |  | 7.689 (1.053-56.170) |  | 10.953 (1.351-88.831) |  |
| N stage | | | | | | | | |
| N0 | 1.000 | *0.046* | 1.000 | 0.066 | 1.000 | 0.058 | 1.000 | 0.189 |
| N1 | 3.371 (1.280-8.879) |  | 2.975 (0.900-9.835) |  | 2.623 (1.145-6.011) |  | 1.625 (0.610-4.330) |  |
| N2 | 3.863 (1.446-10.320) |  | 0.916 (0.216-3.895) |  | 3.055 (1.317-7.089) |  | 0.551 (0.148-2.059) |  |
| M stage | | | | | | | | |
| M0 | 1.000 | *<0.001* | 1.000 | 0.447 | 1.000 | *<0.001* | 1.000 | 0.066 |
| M1 | 9.194 (4.362-19.381) |  | 1.758 (0.410-7.530) |  | 12.375 (6.136-24.959) |  | 4.732 (0.903-24.801) |  |
| Pathological grading | | | | | | | | |
| Well/moderate | 1.000 | 0.619 |  |  | 1.000 | 0.799 |  |  |
| Poor/anaplastic | 1.483 (0.660-3.331) |  |  |  | 1.190 (0.542-2.612) |  |  |  |
| Unknown | 0.882 (0.119-6.544) |  |  |  | 1.505 (0.358-6.322) |  |  |  |
| Venous invasion | | | | | | | | |
| Negative | 1.000 | *0.003* | 1.000 | 0.105 | 1.000 | *0.014* | 1.000 | 0.547 |
| Positive | 3.629 (1.745-7.544) |  | 2.553 (0.972-6.706) |  | 2.608 (1.363-4.989) |  | 1.246 (0.481-3.227) |  |
| Unknown | 1.720 (0.224-13.229) |  | 4.608 (0.478-44.424) |  | 1.220 (0.163-9.141) |  | 3.312 (0.352-31.163) |  |
| Nervous invasion | | | | | | | | |
| Negative | 1.000 | 0.081 |  |  | 1.000 | 0.123 |  |  |
| Positive | 2.049 (0.916-4.586) |  |  |  | 1.803 (0.852-3.814) |  |  |  |
| No. of LNs dissected | | | | | | | | |
| <12 | 1.000 | 0.615 |  |  | 1.000 | 0.65 |  |  |
| ≥12 | 1.357 (0.413-4.466) |  |  |  | 1.271 (0.451-3.582) |  |  |  |
| CEA (μl/ml) | | | | | | | | |
| ≤5 | 1.000 | *<0.001* | 1.000 | *0.005* | 1.000 | *<0.001* | 1.000 | 0.094 |
| >5 | 3.804 (1.808-8.004) |  | 2.025 (0.795-5.155) |  | 3.840 (1.970-7.484) |  | 1.836 (0.789-4.271) |  |
| Unknown | 18.791 (3.970-88.942) |  | 15.846 (2.919-86.030) |  | 10.529 (2.342-47.342) |  | 5.066 (0.998-25.715) |  |
| Adjuvant chemotherapy | | | | | | | | |
| Yes | 1.000 | *<0.001* | 1.000 | 0.054 | 1.000 | *<0.001* | 1.000 | 0.17 |
| No | 1.403 (0.500-3.936) |  | 4.443(1.112-17.757) |  | 1.302 (0.513-3.305) |  | 3.125 (0.931-10.490) |  |
| Unknown | 9.150 (4.134-20.250) |  | 5.341 (1.092-26.118) |  | 8.334 (4.068-17.071) |  | 2.837 (0.505-15.929) |  |
| TILs-PD-1 | | | | | | | | |
| Low | 1.000 | 0.393 |  |  | 1.000 | 0.253 |  |  |
| High | 0.704 (0.315-1.575) |  |  |  | 0.647 (0.306-1.366) |  |  |  |
| TCs-PD-L1 | | | | | | | | |
| Low | 1.000 | 0.396 |  |  | 1.000 | 0.149 |  |  |
| High | 0.737 (0.364-1.491) |  |  |  | 0.624 (0.329-1.184) |  |  |  |
| a All variables are djusted by Cox proportional hazards models including age, gender, T stage, N stage and M stage. b Italic entries indicate statistical significance. | | | | | | | | |
