# Supplementary figures and images for: Prognostic impact of programed cell death-1 (PD-1) and PD-ligand 1 (PD-L1) expression in cancer cells and tumor infiltrating lymphocytes in colorectal cancer
Source: Mol Cancer. 2016 Aug 24;15(1):55. doi: 10.1186/s12943-016-0539-x (PMC4995750; doi:10.1186/s12943-016-0539-x)

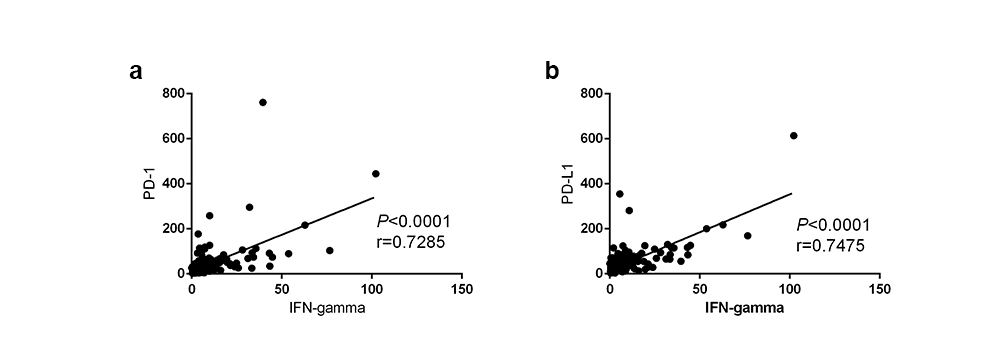

Supplement: Additional file 3: Figure S1. — Correlation of PD-1/PD-L1 and IFN-γ in TCGA database. In TCGA cohort, 250 patients have the data of IFN-γ gene expression. Spearman test was used to determine the correlation of PD-1/PD-L1 and IFN-γ. (a) The expression of PD-1 is directly correlated with IFN-γ (P < 0.0001, r = 0.7285, 95 % CI: 0.6624–0.7833). (b) The expression of PD-L1 is directly correlated with IFN-γ (P < 0.0001, r = 0.7475, 95 % CI: 0.6853–0.7990). (TIF 1066 kb) [file 12943_2016_539_MOESM3_ESM.tif]
